# PET Imaging Biomarkers of Anti-EGFR Immunotherapy in Esophageal Squamous Cell Carcinoma Models

**DOI:** 10.3390/cells7110187

**Published:** 2018-10-27

**Authors:** Tae Sup Lee, In Ho Song, Jong Il Shin, Yong Serk Park, Jung Young Kim, Kwang Il Kim, Yong Jin Lee, Joo Hyun Kang

**Affiliations:** 1 Division of RI Application, Korea Institute of Radiological and Medical Sciences (KIRAMS), Seoul 01812, Korea; inosong@naver.com (I.H.S.); shinjongil@kirams.re.kr (J.I.S.); jykim@kirams.re.kr (J.Y.K.); kikim@kirams.re.kr (K.I.K.); yjlee@kirams.re.kr (Y.J.L.); kang2325@kirams.re.kr (J.H.K.); 2 Department of Biomedical Laboratory Science, College of Health Science, Yonsei University, Wonju 26493, Korea; parkys@yonsei.ac.kr

**Keywords:** EGFR, cetuximab, PET, imaging biomarker, Cu-64, ^18^F-FDG

## Abstract

Epidermal growth factor receptor (EGFR) is overexpressed and considered as a proper molecular target for diagnosis and targeted therapy of esophageal squamous cell carcinoma (ESCC). This study evaluated the usefulness of PET imaging biomarkers with ^64^Cu-PCTA-cetuximab and ^18^F-FDG-PET for anti-EGFR immunotherapy in ESCC models. *In vivo* EGFR status and glucose metabolism by cetuximab treatment were evaluated using ^64^Cu-PCTA-cetuximab and ^18^F-FDG-PET, respectively. Therapeutic responses with imaging biomarkers were confirmed by western blot and immunohistochemistry. TE-4 and TE-8 tumors were clearly visualized by ^64^Cu-PCTA-cetuximab, and EGFR expression on TE-8 tumors showed 2.6-fold higher uptake than TE-4. Tumor volumes were markedly reduced by cetuximab in TE-8 tumor (92.5 ± 5.9%), but TE-4 tumors were refractory to cetuximab treatment. The SUVs in ^64^Cu-PCTA-cetuximab and ^18^F-FDG-PET images were statistically significantly reduced by cetuximab treatment in TE-8 but not in TE-4. ^64^Cu-PCTA-cetuximab and ^18^F-FDG-PET images were well correlated with EGFR and pAkt levels. ^64^Cu-PCTA-cetuximab immuno-PET had a potential for determining EGFR level and monitoring therapeutic response by anti-EGFR therapy. ^18^F-FDG-PET was also attractive for monitoring efficacy of anti-EGFR therapy. In conclusion, PET imaging biomarkers may be useful for selecting patients that express target molecules and for monitoring therapeutic efficacy of EGFR-targeted therapy in ESCC patients.

## 1. Introduction

Esophageal cancer is the sixth leading cause of cancer-related mortality worldwide [1], and it is histologically classified into esophageal squamous cell carcinoma (ESCC) and esophageal adenocarcinoma. ESCC is the major histology in Asian countries, including Japan and China [2,3]. There has been a multidisciplinary approach in surgical techniques, chemotherapy, and radiotherapy treatments used for the condition, with 5-fluorouracil, platinum agents, and taxanes among commonly used agents. However, the outcome for patients with esophageal cancer remains poor [4,5,6,7]. Therefore, novel therapeutic strategies such as molecular-targeted therapy, including small molecule inhibitors of tyrosine kinases (TKIs) and monoclonal antibodies (mAbs), are needed [8,9].

Epidermal growth factor receptor (EGFR, HER-1) overexpression is common in ESCC [10,11]. Previous immunohistochemical studies have shown that 40–50% of ESCC tumors express EGFR [12,13], which is closely associated with disease progression and prognosis [14,15]. Because ESCC patients with high EGFR expression have shown a higher response rate on EGFR-targeting regimen, such as tyrosine kinase inhibitor and anti-EGFR monoclonal antibody, than those with low or moderate EGFR expression [16,17,18,19], EGFR could be a proper molecular target for diagnosis and targeted therapy. Cetuximab, a human–mouse chimerized IgG1 antibody with a high affinity for EGFR, blocks the binding of ligand and induces the internalization and downregulation of EGFR [20]. Antitumor activity of cetuximab has already been reported in preclinical studies [21,22,23] and clinical trials of esophageal cancer [24,25,26].

ESCC patients are usually diagnosed at a later stage because of lack of clinical diagnostic modalities for early diagnosis [27]. In clinical practice, the EGFR status of tumor tissues can be assessed using immunohistochemistry (IHC), which only provides limited information on target expression due to heterogeneity. However, molecular imaging with positron emission radionuclide-labeled monoclonal antibody (mAb), known as immuno-positron emission tomography (immuno-PET), provides noninvasive whole body information on target expressions. Immuno-PET allows visualization and quantification of biodistribution and tumor uptake and thus could be used to predict the efficacy and toxicity of mAb treatment as well as to select individual patients and determine the dosing schedule [28]. Therefore, immuno-PET might provide a valuable strategy for evaluating EGFR expression level in tumor and predicting tumor response to anti-EGFR-targeted therapy.

^64^Cu-labeled cetuximab as an immuno-PET imaging agent has been evaluated in several tumor-bearing mouse models [29,30]. Moreover, ^18^F-fluorodeoxyglucose (FDG) PET has been used for tumor detection and staging [31,32] and monitoring therapy response to anti-HER-1 therapy [33]. However, there have been no reports on predicting the therapeutic efficacy of anti-EGFR therapy using immuno-PET imaging agent.

In this study, we conducted a relationship study between EGFR expression status on immuno-PET imaging and therapeutic effect of cetuximab. We conjugated cetuximab with the bifunctional chelator, 3,6,9,15-tetraazabicyclo[9.3.1]pentadeca-1(15),11,13-triene-3,6,9,-triacetic acid (PCTA) and radiolabeled PCTA-cetuximab with ^64^Cu. We evaluated the in vitro and in vivo feasibility of ^64^Cu-PCTA-cetuximab, an immuno-PET imaging biomarker, in EGFR-expressing ESCC tumor models. We also assessed the changes in *in vivo* EGFR expression level and glucose metabolism by anti-HER-1 therapy using immuno-PET agents ^64^Cu-PCTA-cetuximab and ^18^FDG-PET, respectively, which may provide new strategies in targeted tumor therapy.

## 2. Materials and Methods

### 2.1. Cell Culture

Human ESCC cell lines TE-4 and TE-8 were obtained from RIKEN Bioresource Center Cell Bank (Japan) and grown in RPMI 1640 medium. A431 (human epidermoid carcinoma) and U87-MG (human glioblastoma) were purchased from American Type Culture Collection (Manassas, WV, USA) and maintained in Dulbecco’s Modified Eagle’s medium. All media were supplemented with 10% fetal bovine serum (FBS) and 1% antibiotics/antimycotics. Cultures were maintained at 37 °C in humidified 95% air and 5% carbon dioxide atmosphere.

### 2.2. Reverse Transcription Polymerase Chain Reaction

RNA was extracted using TRIzol (Life Technologies) following the manufacturer’s instructions. Total RNA was reverse-transcripted, and cDNA samples were amplified from PCR reaction mixtures using Onestep RT-PCR kit (Qiagen, Hilden, Germany). The primers used were 5′-cag cgc tac ctt gtc att ca-3′ and 5′-tgc act cag aga gct cag ga-3′ for *EGFR* [34] and 5′- agg tcg gag tca acg gat ttg-3′ and 5′-gtg atg gca tgg act gtg gt-3′ for *GAPDH*.

### 2.3. Immunoblot Analysis

Cell lysates or tumor lysates were subjected to SDS-PAGE, and the protein bands in the gel were transferred to PVDF membranes (Bio-Rad Laboratories, Hercules, CA, USA). The membranes were incubated primary antibodies (EGFR, phosphorylated EGFR, Akt, phosphorylated Akt, and β-actin, Cell Signaling Technology, Danvers, MA, USA) followed by horseradish peroxidase (HRP)-conjugated secondary antibodies (Santa Cruz Biotechnology, Dallas, TX, USA), and the immunoreactive bands were visualized using a chemiluminescent substrate (Thermo Scientific, Waltham, MA, USA).

### 2.4. Flow Cytometry

Cells were incubated with cetuximab (Merck Serono, Darmstadt, Germany) or the isotype control (Rituximab, Roche, Basel, Swiss) antibody for 1 h at 4 °C. After washing twice with phosphate-buffered saline (PBS) containing 1% bovine serum albumin (BSA), the cells were incubated with FITC-conjugated anti-human Ig (Sigma-Aldrich, St. Louis, MO, USA)) for 1 h at 4 °C. Stained cells were analyzed for antibody binding using FACS Calibur (BD Immunocytometry System, San Jose, CA, USA) and CellQuest software (BD Immunocytometry System).

### 2.5. Preparation and Characterization of ^64^Cu-PCTA-Cetuximab

Cetuximab was buffer exchanged and concentrated to 10 mg/mL in 0.1 M sodium bicarbonate buffer pH 8.5 using Vivaspin 2 ultracentrifugation tubes (Sartorius, Göttingen, Germany). A 10-fold molar excess of p-SCN-Bn-PCTA over antibody in DMSO was added to the antibody in 0.1 M sodium bicarbonate buffer pH 8.5. Conjugation was allowed to proceed at room temperature (RT) for 2 h and continued overnight at 4 °C. Unconjugated chelator was removed using Slide-A-Lyzer Dialysis Cassettes (20K MWCO, Thermo Scientific). The immunoconjugate was buffer exchanged and finally concentrated to 2 mg/mL in 20 mM sodium acetate buffer pH 6.5. 

^64^Cu was produced at KIRAMS by 50 MeV cyclotron irradiation using methods previously reported [35]. ^64^CuCl_2_ was prepared in 1 M sodium acetate buffer pH 6.5 and incubated with PCTA-conjugated cetuximab for 60 min at RT. Quality control was performed by instant thin-layer chromatography silica gel (ITLC-SG, Pall, Nassau County, NY, USA) with a mobile phase of 20 mM citrate buffer pH 5 with 50 mM EDTA.

### 2.6. In Vitro Cell Binding Assay

Cell binding studies using ^64^Cu-cetuximab were performed using A431, U87MG, TE-4, and TE-8 cells [35]. Nonspecific binding was determined in the presence of 100-fold excess of cetuximab. After incubation, the samples were washed twice in cold PSA containing 1% BSA. Each sample was counted in a gamma counter (Wizard 1480; Perkin-Elmer, Waltham, MA, USA). Each cell-bound radioactivity (%) was calculated using (cell-bound radioactivity-nonspecific binding radioactivity)/total radioactivity ×100, and data were expressed as relative cell-bound radioactivity using A431 cell-bound radioactivity as a control.

### 2.7. Cell Proliferation Inhibition Assay

TE-4 and TE-8 cells were seeded in 6-well plate at 2.5 × 10^4^ cells per well and incubated for 18 h. Cells were treated with 0–50 μg/mL of cetuximab in RPMI-1640 medium with 2% FBS. After 5 days of incubation, viable cells were counted in a cell counter (ADAM automated cell counter, Digital-Bio, Seoul, Korea). Data were expressed as the percentage of control proliferation using the number of living cells incubated without cetuximab as a control.

### 2.8. Immunotherapy

All animal experiments were done under a protocol approved by KIRAMS Institutional Animal Care and Use Committee (kirams2015-0050). When the tumor volume reached 100–200 mm^3^ (3–4 weeks after inoculation), mice (n = 6 or 7/group) were intravenously administered with cetuximab or isotype antibody (50 mg/kg) twice per week for 4 weeks. Tumor volume was calculated by long diameter × (short diameter)^2^/2, and body weight were measured thrice a week.

### 2.9. Small Animal PET Imaging

We performed immunotherapy twice in TE4 and TE-8 models. In the first experiment (n = 6 or 7/group), ^64^Cu-PCTA-cetuximab was injected 2 days before cetuximab treatment and imaged at 48 h postinjection. After immuno-PET imaging, isotype control or cetuximab were treated to tumor bearing mice. After the first week of immunotherapy, ^64^Cu-PCTA-cetuximab was injected into mice, and immuno-PET images were acquired at 48 h. After treatment for 28 days, mice were sacrificed and tumors were excised, which were used for immunohistochemistry and western blot analysis. In the second immunotherapy treatment (n = 3 or 4/group), ^18^F-FDG-PET imaging was performed before and after cetuximab or isotype control treatment for 3 weeks. ^18^F-FDG-PET imaging was done once per week.

Immuno-PET imaging of tumor-bearing mice was performed using a small animal PET scanner (microPET R4, Concorde Microsystems, Knoxville, TN, USA). ^64^Cu-PCTA-cetuximab (3.0 ± 0.3 MBq, n = 3/group) was injected intravenously into the mice, and static scans were acquired for 60 min at 48 h postinjection. The acquired 3D emission list-mode data were reconstructed for imaging using Fourier rebinning and ordered subsets expectation maximization reconstruction algorithm. Images were visualized using ASIPro display software. We studied no attenuation correction because attenuation for mice is fairly small, and there is very little change in the activity profile across the mice with attenuation correction scan [36]. Quantitative data were expressed as standardized uptake value (SUV), which is defined as tissue concentration (MBq/mL)/injected dose (MBq)/the body weight (g) [35]. The tumor uptake was evaluated from SUV images by 0.5 cm^3^ volume of interest (VOI), which was manually drawn.

^18^F-FDG-PET imaging also used the same instrument. ^18^F-FDG (7.7 ± 0.6 MBq, n = 3/group) was injected intravenously 1 h prior to scan, and static scans were obtained for 20 min. PET images were analyzed and quantified using the abovementioned procedure.

### 2.10. Immunohistochemistry

Following the third dose of isotype or cetuximab, the mice were sacrificed. The tumor tissues were excised, fixed in 4% paraformaldehyde, dehydrated, and embedded in paraffin. Subsequently, terminal deoxynucleotidyl transferase–mediated dUTP nick end labeling (TUNEL) and phosphorylated Akt (pAkt) staining were carried out on tumor sections. In random 6 fields, TUNEL-positive nuclei per field were counted. The pAkt staining index (SI) was defined as the percentage of positive nuclei within the total number of nuclei.

### 2.11. Statistical Analysis

Quantitative data are represented as the mean ± SD, and statistical analysis was performed by one-way ANOVA or Student’s *t* test using GraphPad Prism 5; *p* values of less than 0.05 were considered statistically significant.

## 3. Results

### 3.1. Characterization of EGFR Expression in Esophageal Squamous Cell Carcinoma

**Figure 1:**
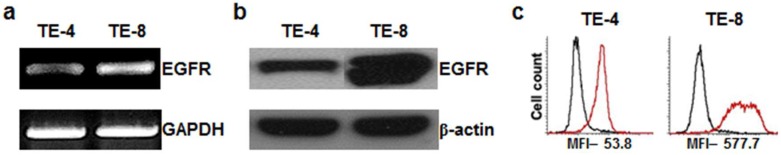
Analysis of epidermal growth factor receptor (EGFR) expression on esophageal squamous cell carcinoma (ESCC) TE-4 and TE-8 cell lines. (**a**) RT-PCR analysis. Internal control used human *GAPDH*. (**b**) Western blot. Internal control used human beta actin. (**c**) Flow cytometry analysis. Black line represents treatment with isotype control and red line represents treatment with cetuximab antibody.

ESCC TE-4 and TE-8 cell lines were examined for *EGFR* expression in RT-PCR, western blot, and flow cytometry in vitro. RT-PCR analysis revealed that *EGFR* mRNA were detectable in TE-4 and TE-8 cell lines (Figure 1a). The primers for *EGFR* and *GAPDH* gene sequence yielded amplification products of the expected size: 195 and 532 bp, respectively. Immunoblot was used to verify the EGFR expression level. EGFR and β-actin bands were detected in TE-4 and TE-8 cell lines (Figure 1b). Flow cytometric analysis (Figure 1c) showed similar results as the western blot data. As determined by western blot and flow cytometry, the TE-8 cell line showed a relatively higher level of EGFR than the TE-4 cell line. TE-8 cells represented higher mean fluorescent intensity (MFI, 577.5) than TE-4 cells (MFI, 53.8).

**Figure 2:**
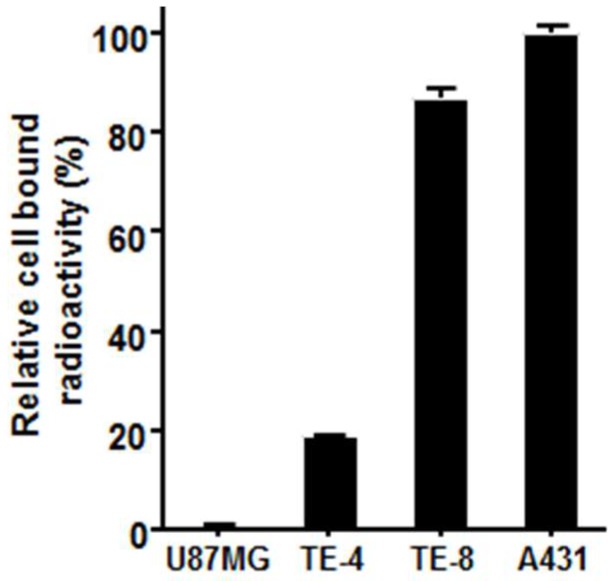
In vitro cell binding assay of ^64^Cu-PCTA-cetuximab. A431 and U87-MG cells were used as positive and negative control, respectively. Data presented as the percentage (%) of relative cell-bound radioactivity to A431 cells.

To determine the cell-surface EGFR expression using ^64^Cu-PCTA-cetuximab, cell binding assay was performed (Figure 2). A431 and U87-MG cell lines were used as a positive and a negative control in this study, respectively. Assuming that EGFR expression level of A431 was 100%, TE-8 cells (87.0 ± 1.8%) had higher EGFR expression, while TE-4 (18.7 ± 0.3%) cells had relatively low EGFR expression. These results indicated that binding of ^64^Cu-PCTA-cetuximab represented the level of EGFR expression on the cells. 

### 3.2. Cytotoxicity of Cetuximab in ESCC Cells

**Figure 3:**
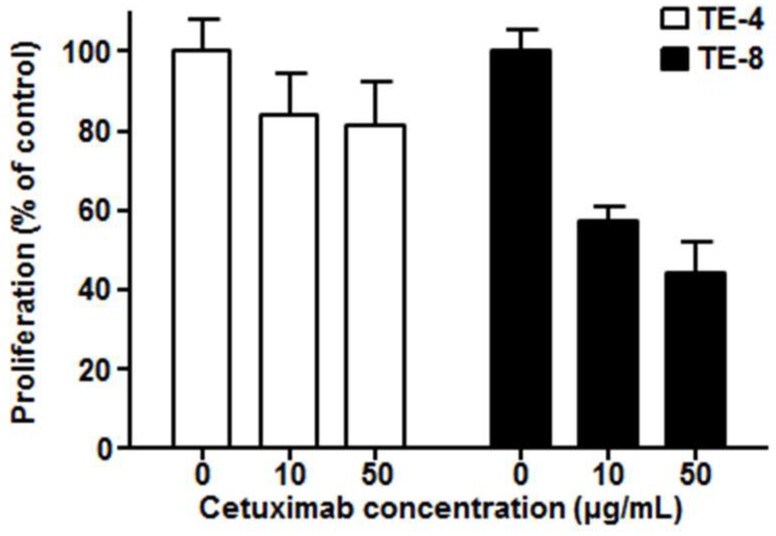
Cytotoxicity of cetuximab on TE-4 (low EGFR expression) and TE-8 (high EGFR expression) cells after five days of cetuximab treatment at each dose. The viable cell number was counted by ADAM cell counter. Data presented as the percentage (%) of viable cell number compared to the control.

We examined the antiproliferative effect of cetuximab against high-EGFR-expressing cell line TE-8 and low-EGFR-expressing cell line TE-4. Cetuximab-induced cell growth inhibition was found in high-EGFR-expressing TE-8 cells, while it was minimal in low-EGFR-expressing TE-4 (Figure 3). TE-8 cells showed 57.2 ± 3.9% (10 μg/mL) and 44.4 ± 7.5% (50 μg/mL) viability compared to control; however, TE-4 cells still kept more than 80% viability even with 50 μg/mL of cetuximab.

### 3.3. Therapeutic Effects of Cetuximab in ESCC Tumors

**Figure 4:**
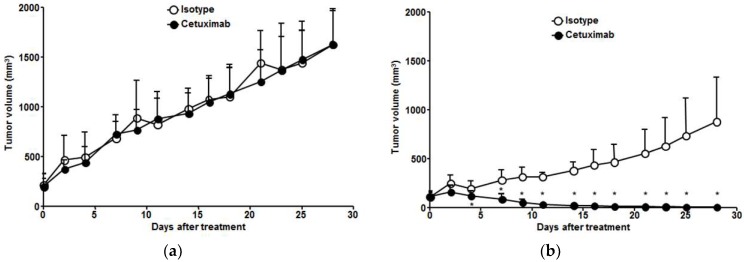
Antitumor effect of cetuximab in ESCC tumor models. Comparison of (**a**) TE-4 and (**b**) TE-8 tumor growth in ESCC xenograft model treated with isotype or cetuximab. Tumor growth in TE-4 was not inhibited by isotype or cetuximab treatment. TE-8 tumor markedly regressed with cetuximab treatment, but TE-8 tumor volume continuously increased with isotype treatment. * Isotype vs. cetuximab, *p* < 0.01.

Antitumor effects of cetuximab were assessed in TE-4 and TE-8 xenograft models (Figure 4). In the isotype group, the growth rate of TE-4 tumors was faster than that of TE-8 tumors. There was no difference in tumor growth between isotype and cetuximab treatment in TE-4 model (Figure 4a). TE-8 tumor growth was inhibited after second administration of cetuximab, and the TE-8 tumor markedly regressed with cetuximab treatment (92.5 ± 5.9% tumor reduction, *p* < 0.001); however, the TE-8 tumor volume continuously increased with isotype treatment (Figure 4b). TE-8 tumor volume in the cetuximab treatment group showed a statistically significant difference after four days (*p* < 0.01). Cetuximab treatment was well tolerated in both TE-4 and TE-8 xenograft models, and no apparent body weight loss was observed (Figure S1).

### 3.4. Characteristics of ^64^Cu-PCTA-Cetuximab

The average number of chelates per cetuximab was determined to be 4.0 ± 0.4 by MALDI mass spectrometry. ^64^Cu-PCTA-cetxuximab were prepared successfully at high radiolabeling yield (>98%) and radiochemical purity (>98%), which were checked by ITLC-SG and size-exclusion HPLC analysis. ^64^Cu-PCTA-cetxuximab had favorable immunoreactive fraction of 0.972, and its radio immunoconjugate showed good in vitro serum stability (above 90%) [35,37].

### 3.5. Immuno-PET Imaging of Cetuximab-Induced Antitumor Activity

**Figure 5:**
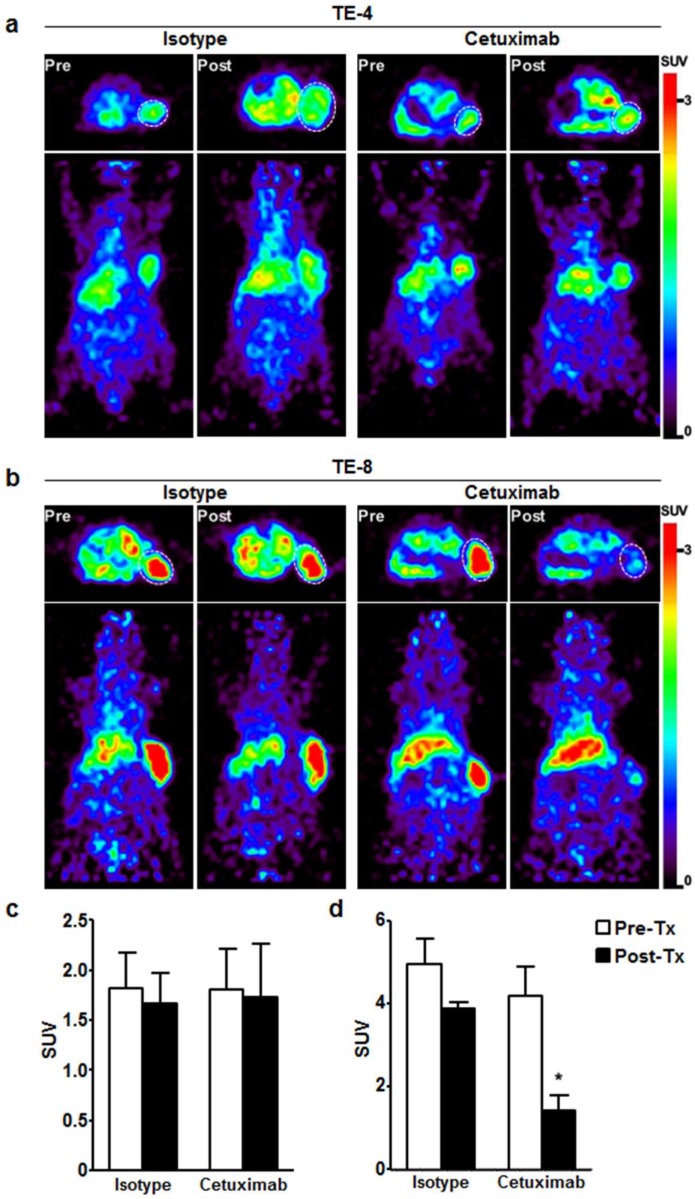
Immuno-PET imaging of EGFR expression level in ESCC xenograft model before and after cetuximab treatment. Transaxial (**Top**) and coronal (**bottom**) PET images of ^64^Cu-labeled cetuximab in (**a**) TE-4 and (**b**) TE-8 tumors determined EGFR level by cetuximab treatment. (**a**,**c**) ^64^Cu-labeled cetuximab uptake was not changed in TE-4 model with isotype or cetuximab treatment. (**b**,**d**) In TE-8 model, ^64^Cu-labeled cetuximab uptake was markedly reduced by only cetuximab treatment. (**c**) and (**d**) Quantitative indices of ^64^Cu-labeled cetuximab in TE-4 and TE-8 tumor represented as standardized uptake value (SUV). * *p* < 0.01. White dotted circle is tumor.

To evaluate the potential of ^64^Cu-PCTA-cetuximab as an immuno-PET imaging agent for determining EGFR level, we performed immuno-PET imaging in TE-4 or TE-8 xenograft models. ^64^Cu-PCTA-cetuximab immuno-PET images (n = 3) were obtained for each animal before treatment and after one week of treatment in TE-4 and TE-8 xenograft models. PET images clearly showed the uptake of ^64^Cu-PCTA-cetuximab in TE-4 and TE-8 tumors at 48 h after injection (Figure 5a,b). The SUV of ^64^Cu-cetuximab before treatment was 2.5-fold higher in TE-8 (4.6 ± 0.7) than TE-4 tumors (1.8 ± 0.3). Immuno-PET images were consistent with the data of flow cytometric assay and cell binding assay. Next, we assessed the tumor response to cetuximab depending on the level of EGFR expression. In TE-4 tumors, there was no statistically significant difference in SUV between pretreated group and post-treated group in isotype-treated (1.8 ± 0.4 vs. 1.7 ± 0.3, *p* = 0.5448) or cetuximab-treated mice (1.8 ± 0.4 vs. 1.7 ± 0.5, *p* = 0.8473) (Figure 5a,c). For TE-8 tumors, there was statistically significant SUV reduction (65.7%) in cetuximab-treated mice compared to isotype control (4.2 ± 0.7 vs. 1.4 ± 0.3, *p* < 0.001) (Figure 5b,d). The SUV of TE-8 tumors in isotype-treated mice seemed to trend downward when compared to the pretreated group; the trend was not significant (*p* = 0.0917). These data indicate that cetuximab-induced antitumor activity could be monitored by ^64^Cu-PCTA-cetuximab, immuno-PET agent, which represents the relative level of EGFR expression in ESCC tumors.

### 3.6. Monitoring Therapeutic Efficacy Using FDG-PET Imaging

**Figure 6:**
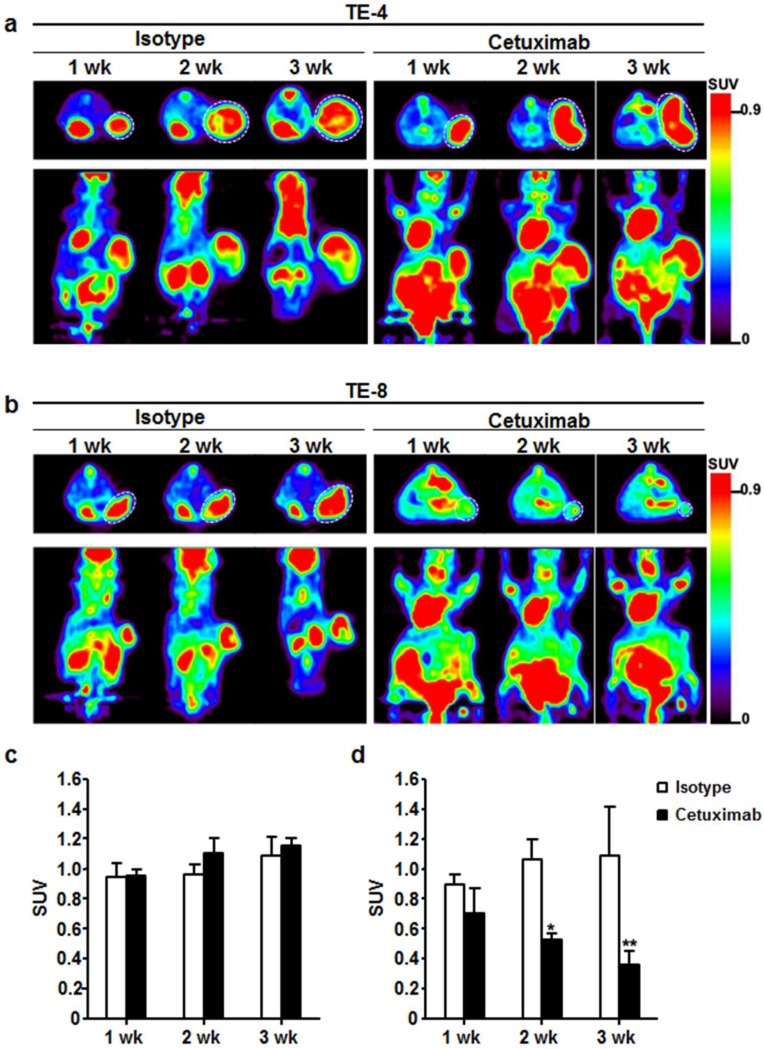
Therapeutic response monitoring with cetuximab treatment using ^18^F-FDG-PET imaging. (**a**) For 3 weeks, ^18^F-FDG uptake was maintained or slightly increased in TE-4 tumor regardless of treatment. (**b**) ^18^F-FDG uptake gradually decreased for 3 weeks in TE-8 tumors by cetuximab treatment. On the other hand, ^18^F-FDG accumulation gradually increased with isotype treatment in TE-8 tumors. (**c**) and (**d**) Quantitative indices of ^18^F-FDG uptake in TE-4 and TE-8 tumor represented as SUV. * *p* < 0.05; ** *p* < 0.01. White dotted circle is tumor.

^18^F-FDG-PET images were obtained once a week for three weeks during treatment. For TE-4 tumors, ^18^F-FDG SUVs in isotype-treated group were 0.9 ± 0.1, 1.0 ± 0.1, and 1.1 ± 0.1 (Figure 6a,c), and those in cetuximab-treated group were 1.0 ± 0.0, 1.1 ± 0.1, and 1.2 ± 0.1 (Figure 6b,d) at 1, 2, and 3 weeks, respectively. Throughout the three-week treatment course, no statistically significant difference in ^18^F-FDG uptake was detected between isotype-treated group and cetuximab-treated group in TE-4 tumors. For TE-8 tumors, the SUVs of ^18^F-FDG in isotype-treated group were 0.9 ± 0.1, 1.1 ± 0.1, and 1.1 ± 0.3, and those in cetuximab-treated group were 0.7 ± 0.2, 0.5 ± 0.0, and 0.4 ± 0.1 at 1, 2, and 3 weeks, respectively. ^18^F-FDG uptake reduction rates in cetuximab-treated group were 21.7%, 49.8%, and 62.4% at 1, 2, and 3 weeks, respectively, compared to ^18^F-FDG uptake in isotype-treated group at each time point. The differences were statistically significant (*p* < 0.01) except for 1 week.

### 3.7. Cetuximab-Induced Apoptosis and Signaling Blockade

**Figure 7:**
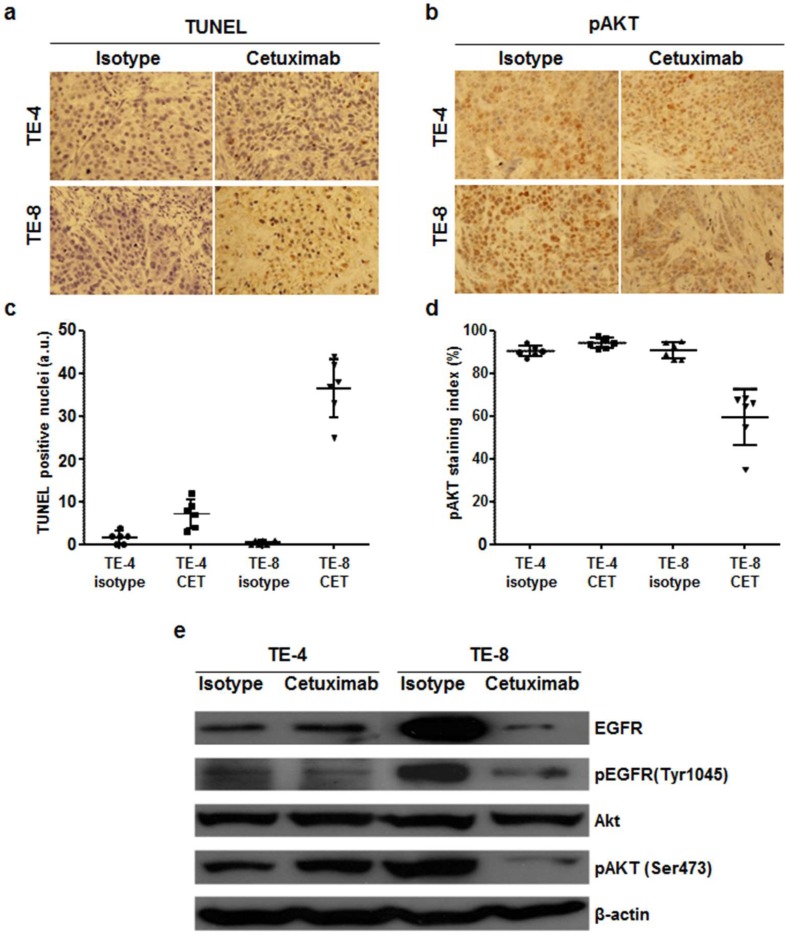
Immunohistochemial staining of TUNEL and phospho-Akt and western blot analysis of TE-4 and TE-8 tumors with isotype or cetuximab treatment. Apoptotic nuclei and phospho-Akt immunoreactivity are shown in brown. (**a**) TUNEL positive nuclei significantly increased in TE-8 tumor with cetuximab treatment. (**b**) pAkt immunoreactivity markedly decreased in cetuximab-treated TE-8 tumor. Representative images were taken under ×400 magnification. (**c**) and (**d**) TUNEL positive nuclei (**c**) and pAkt staining index (**d**) were quantified. (**e**) Western blot analysis of EGFR expression level and its downstream targets in TE-4 and TE-8 tumor. EGFR and pAkt level in TE-8 tumor markedly decreased with cetuximab treatment, but they were maintained in TE-4 tumor.

TUNEL assay was performed to evaluate apoptosis induced by cetuximab treatment in TE-4 and TE-8 tumors. In both tumors, apoptosis was almost unobservable after isotype treatment. TUNEL positive apoptotic cells were more visualized in TE-4 tumor with cetuximab treatment compared to isotype treatment, but it was statistically insignificant (*p* = 0.1085). Apoptotic cells markedly increased in cetuximab-treated TE-8 tumors compared to isotype (*p* < 0.001), indicating that cetuximab treatment increased apoptosis in high-EGFR-expressing TE-8 tumor (Figure 7a,c). pAkt immunoreactivity was significantly reduced in TE-8 tumor with cetuximab treatment compared to isotype (Figure 7b,d). In TE-4 tumors, pAkt positivity slightly increased with cetuximab treatment compared to isotype; however, the difference was nonsignificant (*p* = 0.8614). pAkt immunohistochemical staining result was consistent with western blot analysis of excised tumors. Expression level of EGFR, pEGFR, and pAkt in cetuximab-treated TE-8 tumor markedly decreased compared to isotype-treated group. Exceptionally, pEGFR and pAkt expression level in TE-4 tumor slightly decreased and increased, respectively, with cetuximab treatment. pAkt immunohistochemical staining and western blot results suggested that cetuximab treatment inhibited the PI3K/Akt pathway on TE-8 cells.

## 4. Discussion

In the current study, therapeutic efficacy using cetuximab immunotherapy in ESCC model was associated with EGFR expression level measured by ^64^Cu-cetuximab immuno-PET imaging. Volumetric reduction and metabolic ^18^F-FDG uptake change were evaluated in high-EGFR-expressing TE-8 tumor model. Based on these results, we note that ^64^Cu-PCTA-cetuximab immuno-PET imaging may be useful for evaluating the level of EGFR expression on ESCC tumors in vitro and in vivo and determining change of EGFR expression with cetuximab treatment. ^18^F-FDG-PET was also useful for assessing the therapeutic response to cetuximab treatment in ESCC tumors. Immuno-PET can provide noninvasive, quantitative assessment of specific molecular targets, predict whether the patients are likely to benefit from targeted therapy, and also permit serial monitoring of therapeutic efficacy in the whole level [38].

The receptor expression level alone does not necessarily predict the therapeutic outcome due to lack of information about functional state of the receptor. Thus, pharmacodynamic (PD) biomarker is needed for early identification of responders by quantification of molecular and functional effects and prediction of therapeutic outcomes. Our data indicated that ^64^Cu-PCTA-cetuximab uptake in TE-8 tumors was significantly reduced (65.9% SUV reduction) after one week of cetuximab treatment compared to baseline, suggesting ^64^Cu-PCTA-cetuximab may be useful as an early-phase imaging biomarker for monitoring changes in EGFR expression level (Figure 5c,d). Decreased EGFR expression was also detected by western blot (Figure 7e). To our knowledge, this is the first report to validate the change in EGFR expression in ESCC tumor models with cetuximab treatment. 

Cetuximab inhibits activation of intracellular signaling, including the Ras-Raf-MAPK and phosphoinositide 3-kinase (PI3K)/Akt pathway that are involved in cell growth, survival, and glucose transport [39]. Thus, we postulated that changes in ^18^F-FDG uptake could indicate modulation of EGFR function. ^18^F-FDG-PET showed 21% reduction in the SUV of TE-8 tumors after one week of cetuximab treatment. After three weeks of cetuximab treatment, ^18^F-FDG uptake in TE-8 tumors decreased by 66.8% compared to the isotype treatment. These changes were correlated with the reduction in tumor volume (Figure 5b) and pAkt signal intensity (Figure 7e) of TE-8 tumor. These data are also in accordance with the results reported by Niu et al., who presented a significant decrease in ^18^F-FDG uptake after five days of cetuximab treatment in SSC1 xenografts [40]. Berger et al. also evaluated metabolic response during cetuximab therapy in correlation with clinical response in metastatic colorectal cancer using ^18^F-FDG-PET/CT [41]. On the other hand, preclinical studies using breast cancer cell line for anti-HER2 therapy have shown no significant difference in ^18^F-FDG metabolism following treatment with Hsp90 inhibitor 17-AAG [42] and trastuzumab [43]. Targeted therapy may not always have any observable effect on ^18^F-FDG uptake; however, this study shows the feasibility of ^18^F-FDG-PET as an early PD biomarker of anti-HER1 therapy in ESCC xenograft models.

As shown in in vitro cytotoxicity assay (Figure 3), TE-4 tumor growth was not affected by cetuximab treatment in vivo (Figure 2). The flexibility of tumors to use alternative receptors to activate downstream signaling pathways regulating cetuximab resistance has been reported [44,45,46]. Wheeler et al. reported that acquired resistance to cetuximab reflected dysregulation of EGFR internalization/degradation and subsequent EGFR-dependent activation of HER2 and HER3 in non-small cell lung cancer cell line. Similarly, Yonesaka et al. demonstrated that activation of HER2 signaling led to persistent extracellular signal-regulated kinase 1/2 signaling and consequently to cetuximab resistance. In western blot assay, TE-4 cell is HER2 expressing cell line (data not shown) and pAkt expression level in TE-4 tumor slightly increased with cetuximab treatment compared to isotype treatment (Figure 7b,e). Therefore, aberrant HER2 signaling could be used to activate a bypass signaling pathway in TE-4 tumors. However, the relationship between HER2 expression and cetuximab resistance mechanism need to be assessed in further studies. 

Treatment with TKIs, such as gefitinib and lapatinib, has been shown *in vitro* and *in vivo* to inhibit downstream effector pathway and proliferation of EGFR- and/or HER2-overexpressing ESCC [47,48]. Thus, anti-HER2 targeted therapy is also an attractive approach to treat ESCC. Inhibition of HER2 may play a potential role in antiproliferation of tumor, and inhibition of EGFR and combination therapy with TKIs could be useful in EGFR- or HER2-overexpressing ESCC.

There were some limitations to the current study. First, because two ESCC cell line model was used, expanded preclinical and clinical research are required in various ESCC animal models and clinical patients with tumors expressing varying level of EGFR to validate the association between ^64^Cu-PCTA-cetuximab accumulation in tumor lesion and prognosis after cetuximab immunotherapy and to establish PET signal cutoff value for target selection as imaging biomarker. Second, analysis of genetic mutation of EGFR in ESCC was absent. Although driver gene mutations have not been detected in ESCC, the somatic mutation rate in ESCC is relatively high compared to other solid tumors, and it may attenuate the therapeutic effect to EGFR-targeted therapy [49]. Third, the possibility of the low uptake of ^64^Cu-PCTA-cetuximab in TE-8 model by cetuximab immunotherapy exists. However, there was no reduced uptake of ^64^Cu-PCTA-cetuximab in TE-4 tumor with intermediate EGFR expression by cetuximab treatment (Figure 5a,c). Luo et al. [50] reported systemic pharmacokinetic data of cetuximab in preclinical EGFR-expressing tumor model and nude mice without tumor. The plasma half-life of cetuximab at 1 mg dose of intravenous injection was 39.6 h, *T*_max_ was 3 h, and *C*_max_ was 407.6 g/mL in nude mice without tumor. However, the plasma concentration of cetuximab at 1 mg dose of intraperitoneal injection was 153.6 g/mL at 6 h and 17.7 g/mL at 24 h in tumor-bearing mice. In case of tumor-bearing mice, plasma clearance was faster than nude mice without tumor. Our experiment was also performed in EGFR-expressing tumor. Therefore, the plasma clearance of i.v.-injected cetuximab for treatment would have shown similar patterns as the previous study. Furthermore, TE-4 tumor has moderate EGFR expression, which is easily blocked by treatment dose of cetuximab. However, in our experimental design, TE-4 tumor uptake of ^64^Cu-PCTA-cetuximab was maintained in cetuximab-treated TE-4 tumor at a similar level as that before cetuximab treatment. 

In conclusion, this study shows that ^64^Cu-PCTA-cetuximab immuno-PET and ^18^F-FDG-PET can be used as potential pharmacodynamic PET imaging biomarkers for the therapeutic response to cetuximab treatment in ESCC tumors. ^64^Cu-PCTA-cetuximab immuno-PET imaging biomarker may be useful for selecting patients that express target molecules and monitoring therapeutic efficacy of molecular targeted therapy in clinical trial.

## Supplementary Materials

The following are available online, Figure S1: Relative bodyweight changes with cetuximab treatment in ESCC tumor models.
