# Supplementary material for: PET Imaging Biomarkers of Anti-EGFR Immunotherapy in Esophageal Squamous Cell Carcinoma Models
Source: Cells. 2018 Oct 27;7(11):187. doi: 10.3390/cells7110187 (PMC6262544; doi:10.3390/cells7110187)
Supplement: Supplementary file 1 [file cells-07-00187-s001.pdf]

Article

PET Imaging Biomarkers of Anti-EGFR Immunotherapy in Esophageal Squamous Cell Carcinoma Models

Tae Sup Lee[1,*], In Ho Song[1], Jong Il Shin[1], Yong Serk Park[2], Jung Young Kim[1], Kwang Il Kim[1], Yong Jin Lee[1], Joo Hyun Kang[1]

1 Division of RI Application, Korea Institute of Radiological and Medical Sciences (KIRAMS), Seoul, Republic of Korea; nobelcow@kirams.re.kr (T.S.L); inosong@naver.com (I.H.S); shinjongil@kirams.re.kr (J.I.S); jykim@kirams.re.kr (J.Y.K); kikim@kirams.re.kr (K.I.K); yjlee@kirams.re.kr (Y.J.L); kang2325@kirams.re.kr (J.H.K);

2 Department of Biomedical Laboratory Science, College of Health Science, Yonsei University, Wonju, Republic of Korea; parkys@yonsei.ac.kr (Y.S.P)

* Correspondence: nobelcow@kirams.re.kr ; Tel.: +82-2-970-1351

Supplementary data

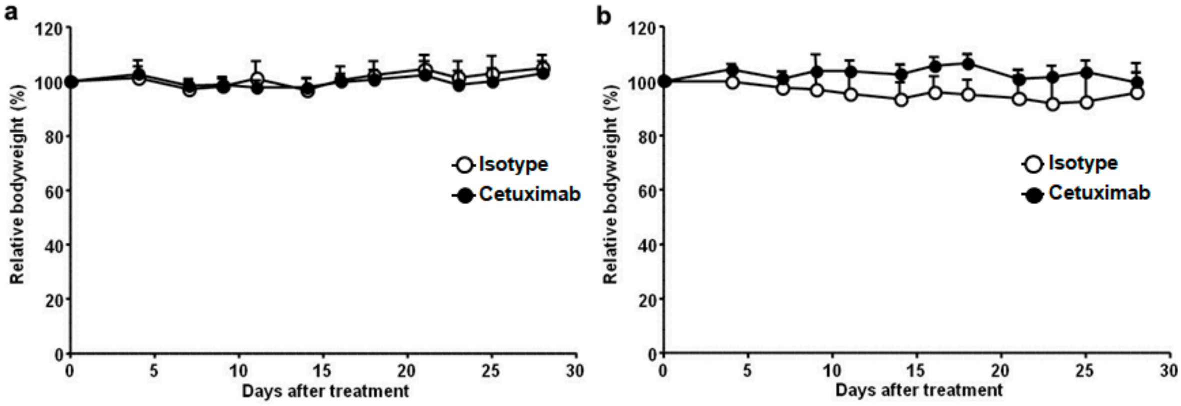

Figure S1. Relative bodyweight changes by cetuximab treatment in ESCC tumor model. a and b, comparison of relative body mass of TE-4 (a) and TE-8 (b) xenograft models treated with isotype or cetuximab. No apparent body weight loss was observed in all groups.

© 2018 by the authors. Submitted for possible open access publication under the terms and conditions of the Creative Commons Attribution (CC BY) license (http://creativecommons.org/licenses/by/4.0/).
